# Molecular-Based Detection of Vector-Borne Diseases in Shelter Dogs in Northern of Vietnam

**DOI:** 10.3390/pathogens15070702

**Published:** 2026-07-02

**Authors:** Bach Xuan Pham, Linh Khanh Bui, Tawin Inpankaew

**Affiliations:** 1 Department of Parasitology, Faculty of Veterinary Medicine, Kasetsart University, Bangkok 10900, Thailand; bachxuan.p@ku.th; 2 Graduate Program in Animal Health and Biomedical Sciences, Faculty of Veterinary Medicine, Kasetsart University, Bangkok 10900, Thailand; 3 Department of Parasitology, Faculty of Veterinary Medicine, Vietnam National University of Agriculture, Hanoi 12406, Vietnam; bklinh5@gmail.com

**Keywords:** canine vector-borne diseases, shelter dogs, molecular detection, Vietnam

## Abstract

Canine vector-borne pathogens (CVBPs) pose a major challenge in shelter medicine, yet data from shelter populations in Vietnam remain unknown. This study determined the prevalence, and risk factors of CVBPs in shelter dogs in northern Vietnam. Blood samples from 300 apparently healthy dogs from three shelters in Hanoi were screened by PCR for *Babesia vogeli*, *Hepatozoon canis*, *Rickettsia* spp., and *Mycoplasma* spp. Representative positive amplicons underwent Sanger sequencing and BLAST analysis. Sequence analysis showed 96.07–100% identity with reference strains, with phylogenetic trees confirming clustering within *B. vogeli*, *H. canis*, *M. haemocanis* and *R. felis* clades. Overall, 43.7% (131/300) of dogs were infected with at least one pathogen, with shelter-level prevalence ranging from 38.0 to 52.0%. Single infections accounted for 35.0%, dominated by *R. felis* (25.7%) and *M. haemocanis* (24.0%), *B. vogeli* and *H. canis* were low (1.3% each). Co-infections were found in 8.7% of dogs, primarily *R. felis* and *M. haemocanis* (8.3%). No evaluated host factors (age, sex, breed, body size, housing style) significantly associated with infection (*p* > 0.05). This study provides the first molecular evidence of canine vector-borne pathogen circulation in Vietnamese shelter dogs, emphasizing the need of ectoparasite control and One Health-oriented surveillance.

## 1. Introduction

Canine vector-borne diseases (CVBDs) have emerged as a significant challenge in shelter medicine as they compromise animal welfare, increase mortality, and complicate rehoming plans for shelter dogs. The term CVBDs refers to a wide range of infections, ranging from bacteria, protozoa, viruses and helminths, which are transmitted via blood meals or the ingestion of infected arthropod vectors [1]. Infections caused by canine vector-borne pathogens (CVBPs) result in numerous non-specific clinical signs, including fever, anorexia, lethargy, weight loss, and pale mucous membranes. Co-infection of multiple vector-borne pathogens has been proved to exhibit more complicated and overlapping clinical signs, further challenging the accurate diagnosis [1].

Shelter-housed dogs are considered a particularly vulnerable population for CVBDs because they often originate from free-roaming backgrounds, which leads to more frequent exposure to ticks, fleas, and other vectors [2]. The shelter environment possesses distinct characteristics, including high animal density and limited antiparasitic measures, which can lead to arthropod vector proliferation and pathogen transmissions [2,3]. In such settings, undetected infections may spread rapidly, resulting in increased morbidity, higher mortality, and additional diagnostic and treatment costs for shelter management. Previous studies in shelter dogs from different regions have indicated high exposure rates to vector-borne pathogens including bacteria and protozoa, highlighting shelters as important foci of infection and reservoirs of zoonotic diseases [4,5,6].

Vietnam, which is a country situated in Southeast Asia (SEA), is geographically divided into three regions: Northern, Central, and Southern. The subtropical climate in northern Vietnam has four distinct seasons, including spring, summer, autumn, and winter. This region is characterized by high average humidity and temperature, providing an ideal ecological condition for the year-round activity of arthropod vectors. The brown dog tick (*Rhipicephalus sanguineus* sensu lato) and fleas (*Ctenocephalides* spp.) have been identified as the most prevalent vectors in Vietnam and the surrounding region [7,8]. These ectoparasites are responsible for the transmission of numerous CVBPs, including *Anaplasma* spp., *Ehrlichia* spp., *Babesia* spp., *Hepatozoon canis*, *Mycoplasma* spp., *Rickettsia* spp. [9]. While recent studies have demonstrated high prevalence of CVBPs in Vietnamese companion dogs and their associated ectoparasites [10,11,12], there are currently no molecular data describing CVBP infection patterns in shelter dogs, and the epidemiological role of shelters in Vietnam remains largely undefined. To address this gap, the present study investigated the molecular prevalence, co-infection patterns, and host-related risk factors of CVBDs in shelter dogs in northern Vietnam. By characterizing CVBPs in this high-density dog population, this study provides shelter-specific baseline data that are critical for designing targeted preventive protocols and for mitigating the spread of zoonotic agents to both human and animal populations.

## 2. Materials and Methods

### 2.1. Research Location and Animals

The sample size was calculated using an infinite population to estimate the prevalence of CVBPs. Based on the previous literature [10], the expected prevalence (p) was estimated at 80% (0.8) and the complementary probability (q) was 20% (0.2). With a confidence level of 95% (z = 1.96) and a desired margin of error (d) of 5% (0.05), the required sample size was calculated as follows:

$$n=\frac{z^{2}pq}{d^{2}}=\frac{1.96^{2}\times 0.8\times 0.2}{0.05^{2}}\approx 246$$

**Figure 1:**
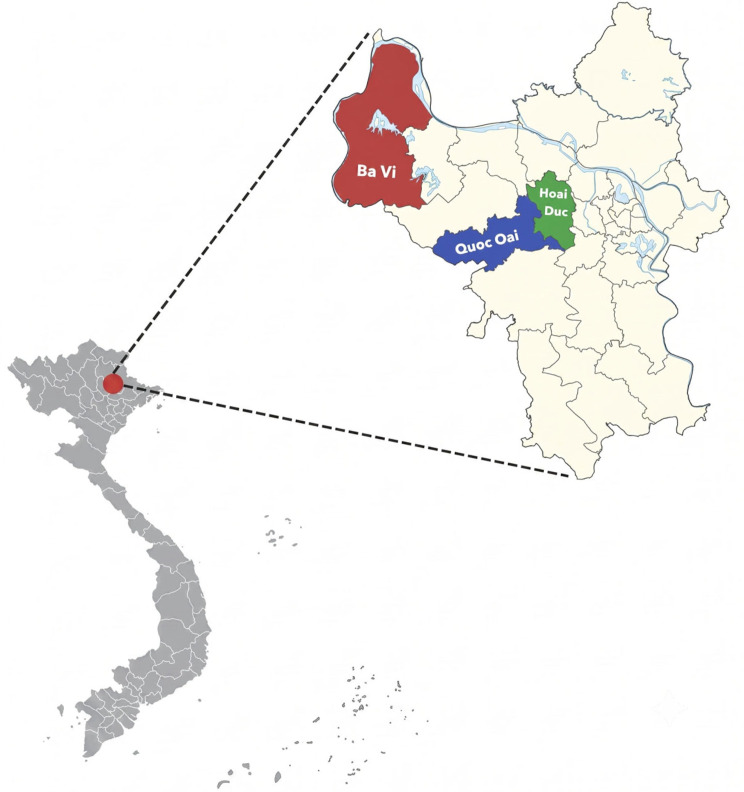
Map of study locations. Main map: Canva Pro. Inset map of Hanoi: Adapted from Wikimedia Commons (by Mai Ngoc Xuan, CC BY-SA 4.0).

A total of 300 blood samples of apparently healthy shelter dogs were randomly collected by cluster sampling method from three shelters in Ba Vi (*n* = 150), Quoc Oai (*n* = 75), and Hoai Duc (*n* = 75) districts, located within Hanoi city, Vietnam (Figure 1). Sample collection was conducted in January 2025. Dogs involved in this study were humanly restrained by experienced veterinarians. For each dog, 2 mL of blood was collected from the cephalic or jugular vein, and transferred to an EDTA tube and kept at 4 °C until used for DNA extraction. Data from participating dogs were collected and categorized, including age (<1 year, 1–5 years, >5 years, defined as puppies, juveniles, adults), breed (domestic, exotic, mixed), sex (male or female), body size (small, medium, large, considered as <10 kg, 10–20 kg, >20 kg), housing style (single-caged or multiple-caged).

### 2.2. DNA Extraction and Molecular Diagnosis

A 200 µL amount of blood from each dog was used for DNA extraction using a commercial DNeasy Blood and Tissue Kit (Qiagen, Hilden, Germany), according to the manufacturer’s instructions. The elution volume was reduced to 100 µL and DNA was stored at −20 °C until used. The genomic DNA samples were tested for the presence of *Babesia vogeli*, *Hepatozoon canis*, *Rickettsia* spp., and *Mycoplasma* spp. by conventional PCR. All primers, targeted genes, and expected amplicon sizes for pathogen detection were listed in Table 1. PCR reactions were performed in 20 µL volumes containing 4 µL of HOT FIREPol^®^ Blend Master Mix (Solis Biodyne, Tartu, Estonia), 1 µL of each 10 pmol primer (forward and reverse), 2 µL of template DNA and nuclease-free water to volume. Amplifications were carried out in a Mastercycler Nexus Gradient thermal cycler (Eppendorf, Hamburg, Germany) using the cycling profiles and annealing temperatures specified for each primer set in Table 1, followed by visualization of products on 1.5% agarose gels under UV illumination. To prevent cross-contamination, strict laboratory workflows were maintained. All procedures, including DNA extraction and PCR master mix preparation, were performed in separate rooms. Each area utilized a dedicated set of pipettes. All pipette tips and microcentrifuge tubes were strictly autoclaved and dried prior to use, and work surfaces and equipment were sanitized before and after each session. For each PCR assay, a positive control and a negative control (nuclease-free water) were included.

### 2.3. Sequence and Phylogenetic Analysis

Representative positive samples for each pathogen were selected for sequencing. Positive amplicons were extracted and purified using the MEGAquick-spinTM plus Fragment DNA purification kit (Intron Biotechnology, Seongnam, Republic of Korea). The purified DNA was then sequenced by a service provider (U2Bio Co., Ltd., Bangkok, Thailand) using Sanger sequencing. Chromatograms were visually inspected and edited in FinchTV (version 1.3.0). The resulting sequences were compared with previously reported sequences using the Basic Local Alignment Search Tool (BLAST + 2.17.0) of the U.S. National Center for Biotechnology Information (NCBI) website (https://blast.ncbi.nlm.nih.gov/Blast.cgi, accessed 16 March, 2026) to determine the species of the pathogen/parasite. All newly generated nucleotide sequences obtained in this study were deposited in the GenBank database. For phylogenetic analysis, multiple alignments including our sequences and selected reference sequences from different geographic regions were constructed, and Neighbor-Joining trees were inferred in MEGA 12 with the Kimura-2-parameter model and 1000 bootstrap replicates. GenBank accession numbers and countries of origin of reference sequences are indicated in the phylogenetic trees.

### 2.4. Statistical Analysis

The infection rates and 95% confidence interval (95% CI) were calculated using Microsoft Excel. Univariate analysis (Chi-square test) was performed to assess the relationship between dogs’ demographic data and the prevalence of infection. The significance level and the confidence interval in the study were set at *p* < 0.05 and 95%, respectively. Statistical analysis was performed using IBM SPSS Statistics (Version 27.0).

## 3. Results

### 3.1. Demographic Data of Shelter Dogs Involved in the Study

The characteristics of the study population are summarized in Table 2. A total of 300 dogs were involved in the study, consisting of 183 (61.0%) males and 117 (39.0%) females. Regarding age distribution, the majority of dogs were juveniles (56.0%), followed by adults (25.3%) and puppies (18.7%). Large dogs comprised the majority of the samples at 51.0%, significantly outnumbering small dogs (24.7%) and medium dogs (24.3%). In terms of breed, exotic breeds were the most prevalent category (64.0%), while domestic and mixed breeds accounted for 30.3% and 5.7, respectively. There were 57.0% of dogs that were kept in a single kennel, and 43.0% were housed in shared cages.

### 3.2. The Prevalence of CVBPs in Shelter Dogs

The overall prevalence of CVBPs was 43.7% (131/300), as shown in Table 3. Regarding the occurrence of individual pathogen, *Rickettsia felis* showed the highest prevalence at 25.7% (77/300; 95% CI: 21.1–30.9%), followed closely by *Mycoplasma haemocanis* at 24.0% (72/300; 95% CI: 19.5–29.1%). *Babesia vogeli* and *Hepatozoon canis* were detected at low rates, at 1.3% each (4/300; 95% CI: 0.5–3.4%). Single infections accounted for 35.0% of cases, whereas co-infections were detected in 8.7% of all dogs. The most common concurrent infection was *R. felis* and *M. haemocanis*, which was identified in 25 dogs (8.3%). Among the three shelters involved in this study, Hoai Duc shelter had the highest infection rate, with 52.0% of dogs testing positive, whereas Quoc Oai and Bavi shelters observed lower prevalence rates of 46.7% and 38.0%, respectively. The difference between prevalence of each shelter is not significant (*p* > 0.05).

### 3.3. Risk Factors Associated with CVBP Infections in Shelter Dogs

The associations between host attributes and CVBP infections are presented in Table 4. Older dogs had a higher prevalence compared to younger dogs. Juveniles and adults showed higher odds of infection than puppies (juveniles: OR = 1.69, 95% CI: 0.9–3.17; adults: OR = 1.58, 95% CI: 0.77–3.22). Regarding body size, CVBPs occurred most frequently among medium-sized dogs (52.1%; OR = 1.73, 95% CI: 0.98–3.04) and small dogs (45.9%; OR = 1.35, 95% CI: 0.77–2.37), compared with large dogs (38.6%). Female dogs (50.4%) exhibited higher odds of infection than males (39.3%; OR = 1.57, 95% CI: 0.98–2.50). Mixed-breed dogs had the highest positivity rate (58.8%; OR = 2.13, 95% CI: 0.78–5.85), followed by domestic (48.4%; OR = 1.40, 95% CI: 0.85–2.31) and exotic breeds (40.1%). Dogs housed in shared cages (48.1%; OR = 1.37, 95% CI: 0.86–2.17) also showed a higher infection rate than those kept in single cages (40.4%). However, none of these variables showed a statistically significant association with CVBP infection (all *p* > 0.05).

### 3.4. Phylogenetic Analysis of CVBPs Found in Shelter Dogs

**Figure 2:**
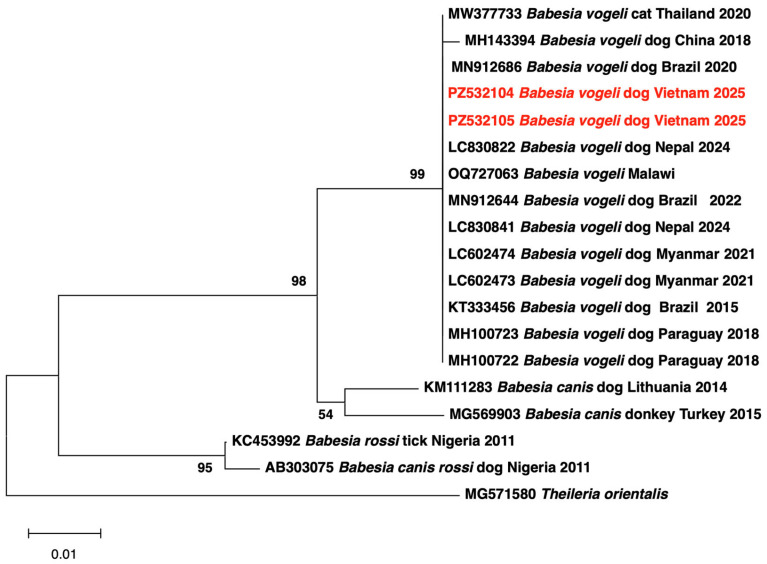
Phylogenetic analysis of *Babesia vogeli* based on the nucleotide sequences of 18S rRNA gene using the Kimura 2 parameter model. Isolates obtained from this study are presented in bold and red color.

**Figure 3:**
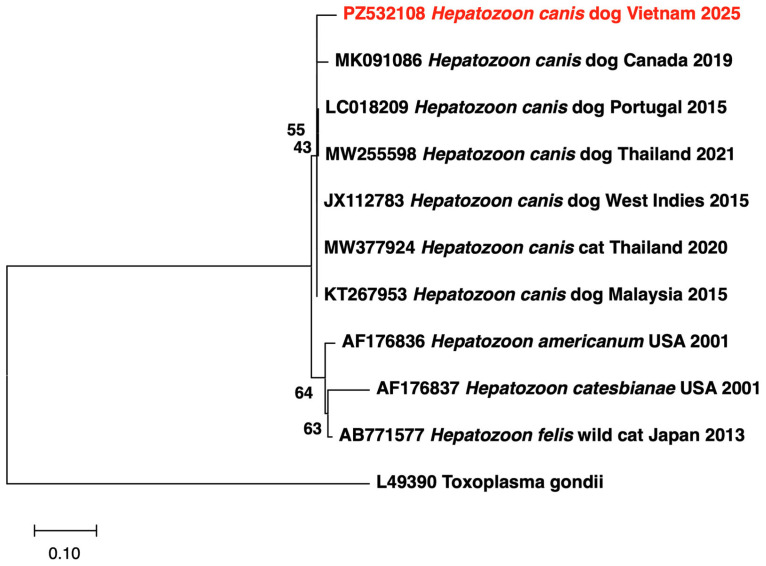
Phylogenetic analysis of *Hepatozoon canis* based on the nucleotide sequences of 18S rRNA gene using the Kimura 2 parameter model. Isolates obtained from this study are presented in bold and red color.

**Figure 4:**
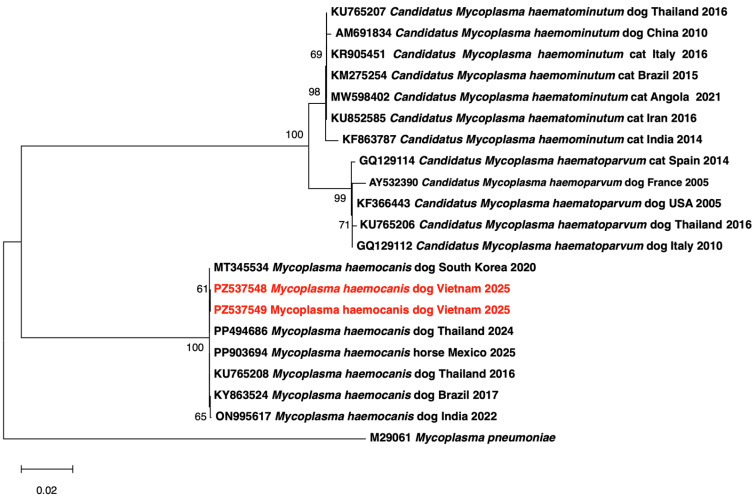
Phylogenetic analysis of *Mycoplasma haemocanis* based on the nucleotide sequences of 16S rRNA gene using the Kimura 2 parameter model. Isolates obtained from this study are presented in bold and red color.

**Figure 5:**
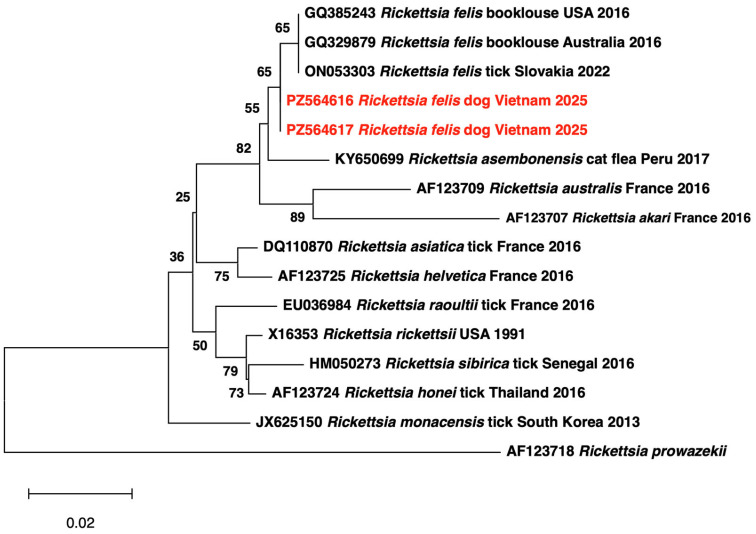
Phylogenetic analysis of *Rickettsia felis* based on the nucleotide sequences of ompB gene using the Kimura 2 parameter model. Isolates obtained from this study are presented in bold and red color.

Sequence analysis of representative amplicons revealed high sequence identity between the pathogens detected in this study and previously reported sequences in the GenBank database, ranging from 96.07% to 100%. The 18S rRNA sequences of positive amplicons for *B. vogeli* showed 100% homology with MH100717, while *H. canis* demonstrated 96.07% similarity with MW810626. Moreover, the 16S rRNA sequences of *Mycoplasma* spp. were 99.63% identical to the *M. haemocanis* sequence MT345534. The obtained ompB sequences for *Rickettsia* spp. showed 99.66% similarity with *R. felis* (ON053303). Phylogenetic trees based on these loci (18S rRNA for *B. vogeli* and *H. canis*, 16S rRNA for *M. haemocanis* and ompB for *R. felis*) grouped all shelter-dog isolates within the corresponding *B. vogeli*, *H. canis*, *M. haemocanis* and *R. felis* clades, with high bootstrap support, and showed close relationships to strains previously reported from other countries (Figure 2, Figure 3, Figure 4 and Figure 5). The representative sequences obtained from this study have been deposited in Genbank database with the following accession numbers: PZ532104-PZ532105 for *B. vogeli*, PZ532108 for *H. canis*, PZ564616-PZ564617 for *R. felis*, and PZ537548-PZ537549 for *M. haemocanis*.

## 4. Discussion

The overall prevalence of CVBPs found in this study was 43.7% across three shelters in northern Vietnam. This prevalence is comparable to previous studies on stray dogs in Thailand and shelter dogs in Malaysia, where 43.1% and 35.0% of the populations were found to be infected with one or more pathogens, respectively [17,18]. However, other studies of stray and semi-domesticated dogs in the SEA region demonstrated higher prevalence, ranging from 71.3% to 86.5% [19,20,21]. Such discrepancies could be attributed to differences in targeted pathogens, dog populations, and geographic or environmental conditions. It was initially hypothesized that shelter dogs would have a higher rate of infection than companion dogs in Vietnam, largely due to their free-roaming lifestyle before entering the shelter, high vector exposure, and unknown historical antiparasitic prevention [2]. Interestingly, the CVBPs prevalence among shelter dogs in the current study was considerably lower than that found in client-owned dogs (73.9%, 252/341) in the same region in Vietnam [10]. Several reasons could explain for the difference between the prevalence of this study and reported prevalence of Vietnamese dogs. Firstly, sampling was conducted in the cool, dry winter season with an ambient temperature of 12–18 °C, which could reduce activity and reproduction of *Rhipicephalus linnaei* (formerly known as *Rhipicephalus sanguineus* sensu lato) and *Ctenocephalides felis*. In this study, clinical inspection revealed low ectoparasite infestation rate of shelter dogs during sample collection. In contrast, surveys on owned dogs in Vietnam were carried out throughout the year and peak prevalence of CVBPs was observed in warmer months [10,11,12]. Secondly, the three participating shelters administered at least intermittent ectoparasiticide treatment three months prior to this study with ivermectin and dogs were housed in closed kennels, which could reduce tick and flea infestation and CVBP infection. Thirdly, the pathogen panel differed across studies. This study targeted four pathogens, whereas Do et al. [10] additionally screened for *Ehrlichia*, *Anaplasma*, which can inflate their cumulative prevalence.

Phylogenetic results demonstrate that the *B. vogeli*, *M. haemocanis*, and *R. felis* isolates from shelter dogs in northern Vietnam are genetically similar to strains circulating reported in other countries, contributing to the molecular evidence of detected pathogens in shelter populations in Vietnam.

Among the targeted pathogens in this study, the two flea-associated pathogens predominated, including *R. felis* that accounted for 25.7% (95% CI: 21.1–30.9%) and *M. haemocanis* for 24.0% (95% CI: 19.5–29.1%). *R. felis* is maintained primarily in *Ctenocephalides felis* fleas, with dogs acting as incidental or secondary mammalian hosts, whereas *M. haemocanis* is a haemotropic bacterium of dogs that can be transmitted by ectoparasites, including brown dog ticks and possibly fleas, as well as by direct blood exposure such as dog fights or transfusion. In this study, *R. felis* prevalence is consistent with emerging evidence of *R. felis* endemicity in Vietnam. Previously, *R. felis* DNA has been detected in 29.2% of buffy coat samples from household dogs in the Central Highlands of Vietnam, confirming the role of dogs as natural mammalian reservoirs for flea-borne spotted fever in the country [22]. Likewise, 24.0% prevalence of *M. haemocanis* markedly higher than 9.4% reported in companion dogs from northern Vietnam [10] and is comparable to the 27.04% documented in free-roaming dogs in southern Thailand [20]. A study in Malaysia further reported that shelter dogs had a significantly higher *M. haemocanis* prevalence (45.1%) than pet dogs (11.8%) from the same country [23], suggesting that the living conditions and environmental factors within shelters can facilitate transmission via vectors or direct contact. Several biological and ecological features can explain why these two flea-associated pathogens predominated in surveyed dogs. Notably, visual inspection at sampling revealed very few dogs with active ectoparasite infestation. Only a small number of shelter dogs carried ticks and no heavy flea burdens were observed. This apparent mismatch between low ectoparasite infestation at sampling point and high prevalence of *Rickettsia* and *Mycoplasma* could be explained by *C. felis* ecology. The cat flea has a short adult phase relative to a long environmental phase with 95% lifetime and exists as eggs, larvae, and pupae developing in the environment [24]. Therefore, a kennel can sustain continuous transmission pressure without active infestation on the host. In addition, both *R. felis* and haemotropic *Mycoplasma* spp. are often sub-clinical infections in dogs [9]. Molecular positivity observed in this study therefore indicates sub-clinical infections rather than active infection at sampling time. Moreover, the intermittent ectoparasite preventive programs of the shelters are effective at suppressing adult fleas on the host but only partially reduce environmental immature stages, thus dogs may remain positive after visible infestation has been controlled. The high prevalence of emerging bacterial pathogens poses a zoonotic threat to the community. Human non-specific febrile illness caused by *R. felis* has been confirmed by serological and molecular assays in some SEA countries, including Laos, the Thailand–Myanmar border region, and Vietnam [25,26,27]. While the zoonotic relevance of *M. haemocanis* has historically been considered low, a recent molecular investigation has confirmed *M. haemocanis* DNA in humans living in close contact with dogs [28]. This evidence indicates the need for integrated One Health surveillance, including routine diagnosis and ectoparasite control, public education on flea and tick prevention.

In contrast, the prevalence of *B. vogeli* and *H. canis* was detected at a comparatively low rate of 1.3% each (4/300; 95% CI: 0.5–3.4%) in the present study. These findings are substantially lower than those reported for companion dogs in northern Vietnam, where *B. vogeli* was the most frequently detected pathogen at 30.5%, and also lower than the 32.7% *B. vogeli* prevalence recorded in semi-domesticated dogs in Cambodia [10,19]. Similarly, previous studies from neighboring countries have reported *H. canis* prevalence rates from 10.9 to 18.8%, far exceeding the rate found in the current study [16,18]. The low detection rate of the tick-borne protozoa *B. vogeli* and *H. canis* in this study is consistent with a low burden of brown dog ticks in the surveyed shelters, given that *Rhipicephalus linnaei* (formerly *R. sanguineus* sensu lato) is the primary vector for these pathogens [29,30].

Co-infections were detected in 8.7% (26/300) of the tested dogs, with the co-occurrence of *R. felis* and *M. haemocanis* being the most common combination (8.3%, 25/300). In warm regions, the diversity of ectoparasites and vector-borne pathogens has been demonstrated to be associated with a higher co-infection rate in dogs [31,32]. The transmission of multiple pathogens can be facilitated either by a single vector co-infected with several pathogens or by distinct vector species transmitting individual pathogens [31]. In this current study in Vietnam, the diagnosis of CVBPs mainly relies on complete blood count parameters, microscopy, and clinical examinations. However, these conventional methods offer low diagnostic accuracy as the clinical presentations are often more complicated or overlap in co-infection cases. Therefore, this underscores the need for routine molecular diagnosis to screen multiple pathogens to improve diagnostic efficiency and treatment outcomes.

Univariate logistic regression revealed no statistically significant associations between any of the host-related variables (including age, sex, breed, body size, and lifestyle) and CVBP infection (*p* > 0.05). While numerous studies have reported significant relationships between dogs’ demographic data and CVBP infections [10,33,34], a study on companion dogs and cats in Thailand revealed only age associated with the infections [35]. All shelter dogs involved in this study had minimal ectoparasite control and the same vector exposure risk across the three shelters, suggesting that environmental and management-level factors, such as ectoparasite burden, and antiparasitic treatment, are more likely to be the primary determinants of infection risk than host demographic characteristics.

Regarding shelter management perspective, the findings of this study offer several critical, practical implications for improving animal welfare and controlling tick and flea-borne diseases. Year-round systemic tick and flea control utilizing long-acting isoxazolines or multi-action imidacloprid/fipronil formulations must be prioritized over traditional, seasonal acaricides to provide continue protection and reduce ectoparasite burdens. Routine environmental treatment of kennels and communal areas is required to eliminate life stages residing within the shelters. In addition, a strict intake protocol should be enforced: all newly admitted dogs should undergo an initial isolation and monitoring period before being introduced into the shelter population. Finally, as the clinical signs of CVBP infections are non-specific, shelters should also adopt PCR-based screening of new arrivals and of any dog presenting with fever, anaemia, or non-responsive lethargy for better diagnostic and treatment efficiency.

Several limitations should be addressed regarding the current study. Firstly, the cross-sectional study design and winter-season sampling period may have underestimated the true prevalence of CVBPs among shelter dogs. Secondly, dog samples were collected from only three major shelters in northern Vietnam, which limits the geographic and demographic representativeness of the findings. Finally, the restriction of the study population to apparently healthy dogs may result in underrepresentation of the prevalence and co-infection patterns presenting in higher-risk or clinically symptomatic animals.

## 5. Conclusions

This study provides the first molecular evidence of canine vector-borne pathogen circulation in shelter dogs in northern Vietnam and identifies shelters as a distinct epidemiological niche for flea-borne infections. Based on the high prevalence, shelter vector-control programs should prioritize long-acting systemic ectoparasite prophylaxis for all dogs, regular environmental treatment of kennels and communal areas, and implementing standardized intake protocols to reduce introduction and spread of CVBPs within shelters. Integration of shelter data into local One Health surveillance, together with staff training and public education on ectoparasite control, is essential to mitigate potential zoonotic transmission risks.

## Figures and Tables

**Table 1:** Sets of primers used in this study.

Pathogen	Primer (F/R) 5′-3′	Product Size (bp)	Target Gene	Annealing Temperature	Reference
*Rickettsia* spp.	CGACGTTAACGGTTTCTCATTCT ACCGGTTTCTTTGTAGTTTTCGTC	252	ompB	54 °C	[13]
*Mycoplasma* spp.	ATACGGCCCATATTCCTACG TGCTCCACCACTTGTTCA	595–618	16S rRNA	60 °C	[14]
*Babesia vogeli*	GTTTATTAGTTTGAAACCCGC GAACTCGAAAAAGCCAAACGA	455	18S rRNA	60 °C	[15]
*Hepatozoon canis*	ATACATGAGCAAAATCTCAAC CTTATTATTCCATGCTGCA	666	18S rRNA	60 °C	[16]

ompB: outer membrane protein B, rRNA: ribosomal Ribonucleic Acid.

**Table 2:** Demographic data of the study population.

Variable	Category	Count	Percentage (%)
Age	<1 year	56	18.7
	1–5 years	168	56.0
	>5 years	76	25.3
Body Size	Small	74	24.7
	Medium	73	24.33
	Large	153	51.0
Sex	Male	183	61.0
	Female	117	39.0
Breed	Domestic	91	30.3
	Mixed	17	5.7
	Exotic	192	64.0
Lifestyle	Single-caged	171	57.0
	Multiple-caged	129	43.0

**Table 3:** Prevalence of CVBP infections in shelter dogs.

Pathogen	Shelter Bavi (*n* = 150)	Shelter Hoai Duc (*n* = 75)	Shelter Quoc Oai (*n* = 75)	Total (*n* = 300)	95% CI
**Total of infections**	**57 ^a^** **(38.0%)**	**39 ^a^** **(52.0%)**	**35 ^a^** **(46.7%)**	**131** **(43.7%)**	**38.2–49.3%**
*Babesia vogeli*	0 (0%)	2 (2.7%)	2 (2.7%)	4 (1.3%)	0.5–3.4%
*Hepatozoon canis*	4 (2.7%)	0 (0%)	0 (0%)	4 (1.3%)	0.5–3.4%
*Rickettsia felis*	36 (24.0%)	19 (25.3%)	22 (29.3%)	77 (25.7%)	21.1–30.9%
*Mycoplasma haemocanis*	25 (16.7%)	27 (26.0%)	20 (26.7%)	72 (24.0%)	19.5–29.1%
**Single infection**	**49** **(32.7%)**	**30** **(40.0%)**	**26** **(34.7%)**	**105** **(35.0%)**	**29.8–40.1%**
*B. vogeli*	0 (0%)	2 (2.7%)	2 (2.7%)	4 (1.3%)	0.5–3.4%
*H. canis*	3 (2.0%)	0 (0%)	0 (0%)	3 (1.0%)	0.3–2.9%
*R. felis*	29 (19.3%)	10 (13.3%)	13 (17.3%)	52 (17.3%)	13.5–22.0%
*M. haemocanis*	17 (11.3%)	18 (24.0%)	11 (14.7%)	46 (15.3%)	11.7–19.9%
**Co-infection**	**8** **(5.3%)**	**9** **(12.0%)**	**9** **(12.0%)**	**26** **(8.7%)**	**6.0–12.4%**
*H. canis* + *M. haemocanis*	1 (0.7%)	0 (0%)	0 (0%)	1 (0.3%)	0.1–1.9%
*R. felis* + *M. haemocanis*	7 (4.7%)	9 (12.0%)	9 (12.0%)	25 (8.3%)	5.7–12.0%

Values within the same row with the same superscript letters (^a^) are not significantly different (*p* > 0.05) based on Chi-square post hoc comparison with Bonferroni correction.

**Table 4:** Univariate analysis of dogs’ demographic data and CVBP infections.

Variables	No. Tested Dogs	No. Positive (%)	χ^2^	*p*-Value	OR (95% CI)
**Age**					
Puppy (<1 year)	56	19 (33.9)	2.715	0.257	Ref.
Juvenile (1–5 years)	168	78 (46.4)			1.69 (0.9–3.17)
Adult (>5 years)	76	34 (44.7)			1.58 (0.77–3.22)
**Body size**					
Large (>20 kg)	153	59 (38.6)	3.865	0.145	Ref.
Medium (10–20 kg)	73	38 (52.1)			1.73 (0.98–3.04)
Small (<10 kg)	74	34 (45.9)			1.35 (0.77–2.37)
**Sex**					
Male	183	72 (39.3)	3.564	0.059	Ref.
Female	113	59 (50.4)			1.57 (0.98–2.50)
**Breed**					
Exotic	192	77 (40.1)	3.390	0.184	Ref.
Mixed	17	10 (58.8)			2.13 (0.78–5.85)
Domestic	91	44 (48.4)			1.40 (0.85–2.31)
**Lifestyle**					
Single-caged	171	69 (40.4)	1.777	0.182	Ref.
Multiple-caged	129	62 (48.1)			1.37 (0.86–2.17)

Ref. = Reference.

## Data Availability

The original contributions presented in this study are included in the article. Further inquiries can be directed to the corresponding author. The datasets generated and analyzed during the current study are available from the corresponding author on reasonable request. All nucleotide sequences generated in this study have been deposited in the GenBank database under the following accession numbers: *Rickettsia felis* ompB gene, PZ564616 and PZ564617; *Mycoplasma haemocanis* 16S rRNA gene, PZ537548 and PZ537549; *Hepatozoon canis* 18S rRNA gene, PZ532108; *Babesia vogeli* 18S rRNA gene, PZ532104 and PZ532105.

## Abbreviations

The following abbreviations are used in this manuscript:

CVBPs	Canine vector-borne pathogens
CVBDs	Canine vector-borne diseases
PCR	Polymerase chain reaction
SEA	Southeast Asia
